# Assessing the depression risk in the U.S. adults using nomogram

**DOI:** 10.1186/s12889-022-12798-6

**Published:** 2022-03-02

**Authors:** Yafeng Zhang, Wei Tian, Xinhao Han, Guangcan Yan, Yuanshuo Ma, Shan Huo, Yu Shi, Shanshan Dai, Xin Ni, Zhe Li, Lihua Fan, Qiuju Zhang

**Affiliations:** 1 Department of Health Management, School of Health Management, Harbin Medical University, No.157 Baojian Road, Harbin, 150081 China; 2 Department of Biostatistics, School of Public Health, Harbin Medical University, No.157 Baojian Road, Harbin, 150081 China; 3 Sichuan Kelun Pharmaceutical Co, No. 36 Baihua West Road, Chengdu, 610071 China; 4 People’s medical publishing house, No. 19 Panjiayuan South Road, Beijing, 100021 China; 5 Beijing Children’s Hospital, Capital Medical University, National Center for Children’s Health, No. 56 Nanlishi Road, Beijing, 100045 China

**Keywords:** Depression, Prediction model, Nomogram

## Abstract

**Background:**

Depression has received a lot of attention as a common and serious illness. However, people are rarely aware of their current depression risk probabilities. We aimed to develop and validate a predictive model applicable to the risk of depression in US adults.

**Methods:**

This study was conducted using the database of the National Health and Nutrition Examination Survey (NHANES, 2017–2012). In particular, NHANES (2007–2010) was used as the training cohort (*n* = 6015) for prediction model construction and NHANES (2011–2012) was used as the validation cohort (*n* = 2812) to test the model. Depression was assessed (defined as a binary variable) by the Patient Health Questionnaire (PHQ-9). Socio-demographic characteristics, sleep time, illicit drug use and anxious days were assessed using a self-report questionnaire. Logistic regression analysis was used to evaluate independent risk factors for depression. The nomogram has the advantage of being able to visualize complex statistical prediction models as risk estimates of individualized disease probabilities. Then, we developed two depression risk nomograms based on the results of logistic regression. Finally, several validation methods were used to evaluate the prediction performance of nomograms.

**Results:**

The predictors of model 1 included gender, age, income, education, marital status, sleep time and illicit drug use, and model 2, furthermore, included anxious days. Both model 1 and model 2 showed good discrimination ability, with a bootstrap-corrected C index of 0.71 (95% CI, 0.69–0.73) and 0.85 (95% CI, 0.83–0.86), and an externally validated C index of 0.71 (95% CI, 0.68–0.74) and 0.83 (95% CI, 0.81–0.86), respectively, and had well-fitted calibration curves. The area under the receiver operating characteristic curve (AUC) values of the models with 1000 different weighted random sampling and depression scores of 10–17 threshold range were higher than 0.7 and 0.8, respectively. Calculated net reclassification improvement (NRI) and integrated discrimination improvement (IDI) showed the discrimination or accuracy of the prediction models. Decision curve analysis (DCA) demonstrated that the depression models were practically useful. The network calculators work for participants to make personalized predictions.

**Conclusions:**

This study presents two prediction models of depression, which can effectively and accurately predict the probability of depression as well as helping the U.S. civilian non-institutionalized population to make optimal treatment decisions.

**Supplementary Information:**

The online version contains supplementary material available at 10.1186/s12889-022-12798-6.

## Background

Depression is a common and debilitating mental health disorder [1]. The syndrome of depression mainly includes either depressed mood or loss of interest or pleasure [2]. According to literature studies, more than 264 million people worldwide are affected by depression [3]. In practice, its detection, diagnosis, and management often pose challenges for clinicians because of its various presentations, unpredictable course and prognosis [4]. Therefore, it is important to develop an effective predictive model for depression risk.

Sociodemographic characteristics such as gender, race, income, education, age and marital status have been reported to be independently associated with depression [5, 6]. It is generally believed that in the gender difference in depression, women experience major depression twice as much as men [7]. Compared to other racial and ethnic groups, even though African Americans experienced more of the stressors, but they showed lower levels of depression [8]. Besides, the lower individual’s income, the more likely they are to face a particularly bleak socio-economic outlook, which is partly linked to depression. In particular, studies have found that the cumulative income of patients with depression only accounts for 51% of the income of the general population, and the unemployment rate is higher [9]. Higher education was associated with a lower risk of future depression throughout the life course [10, 11]. And a study showed that 42% of depression cases had no higher education compared to 27% of the general population [9]. For both male and female, the study found that depression peaks between ages 40 and 50, accompanied by low self-esteem, sleep disorders and other symptoms [12]. It was also found that marriage and other intimate romantic partnerships (e.g., cohabitation) promote mental health and reduce depressive stress by providing social support [13].

The current study also found that sleep quality and quantity are very often substantially decreased in depression [14]. Approximately 80% of depressive states are associated with comorbid insomnia [15]. Similarly, depression and anxiety are highly prevalent psychiatric disorders, with a large overlap in pathophysiology and sharing a high degree of comorbidity [16]. However, anxiety and depression were more connected within-disorder than between-disorders, with anhedonia, sad mood, and worry is the bridge between depression and anxiety symptoms [17]. And studies have found that illicit drug use (ie, cannabis, cocaine and heroin) can increase the risk of developing depressive symptoms and mood disorders [18]. Although the above factors have been found to be associated with depression, their applicability in assessing the depression risk in the population remains to be determined.

Currently, the Structured Clinical Interview for Diagnostic and Statistical Manual of Mental Disorders is considered the gold standard for rating depression, and the PHQ-9 is widely used as a validated tool for population screening for depression. Although there have been significant advances in understanding the pathophysiology of depression, there is still no or limited biological evidence to support the decision, misdiagnosis and underdiagnosis are common problems [19]. And depression is vulnerable to prejudice and discrimination. It was found that few studies have developed practical predictive tools to examine the risk of depression in populations. Of all the available models, the nomogram can provide an individualized, evidence-based, highly accurate risk estimation. Therefore, the purpose of this study was to develop a depression risk assessment model appropriate for the U.S. adult population to assist and identify at-risk populations.

## Methods

### Study design and participants

The National Health and Nutrition Examination Survey (NHANES) is a program of studies designed to assess the health and nutritional status of the population in the United States. NHANES uses a complex, multistage, probability sampling design, with an average of about 5000 people surveyed every 2 years. We cleaned the NHANES data, removed the missing samples, and selected the NHANES (2007–2012) as the raw dataset for our analyses. As shown in eFig. 1 (Data Supplement), the flow diagram of the study participants shows the inclusion and exclusion criteria of the training cohort and validation cohort. In total, the training cohort comprised 6015 participants from the NHANES (2007–2010) data and 2812 participants from NHANES (2011–2012) as the validation cohort. All 8827 participants were adults (age from 20 to 59 years), with a mean (SD) age of 39.5 (11.8) years, 43.9% non-Hispanic-white and 49.7% female. Data Supplement presents other characteristics of the population sample, as well as the training cohort and validation cohort. NHANES has been approved by the National Center for Health Statistics Ethics Review Board. Written informed consent was obtained from all participants [20].

### Measurements

It is well known that the Patient Health Questionnaire (PHQ-9) is a widely used screening tool for non-psychiatric depression [21]. Hence, we used the PHQ-9 scale to assess depression, a 9-item questionnaire that measures depression on a four-point Likert scale (0= “not at all,” 1 = “several days,” 2= “more than half the days”, or 3= “nearly every day”, ranges 0–27), with a depression score of more than 10, usually considered to be suffering from depression. Of course, some studies also believe that cut-off score between 8 and 11 in PHQ-9 has acceptable diagnostic properties for depression [22]. We used an algorithm based on DSM-IV criteria and based on cut-off summed-item scores to defining depression [21]. The algorithm method requires a total of at least five symptoms rated as at least 2 (more than half the days), with the exception of the suicidal ideation item, which counts as one of the five symptoms if it is rated as 1 (several days) or above. The algorithm also requires that at least one of the symptoms scored as at least 2 is either loss of interest or pleasure or depressed mood. Alternatively, a cut-off score of 10 or above on the summed-item score was also diagnosed as depression. Finally, the 10th item was added to the diagnostic part of the PHQ-9 asking patients how difficult the problems identified made it for them to manage work, daily living and relationships. The PHQ-9 has been certified to be an effective measure of detecting depression across major U.S. sociodemographic groups [23, 24].

Information on drug use is collected from the drug use questionnaire (DUQ), including on lifetime and current use of marijuana or hashish, cocaine, heroin, and methamphetamine, as well as intravenous use of drugs. We redefined and classified drug use in the original variables: 1) drug use, defined as a self-report of ever using marijuana or hashish, cocaine, methamphetamines, heroin or injecting the drug in the participant’s lifetime; 2) Illicit drug use, defined as a self-report of ever using cocaine, methamphetamines, heroin or injecting the drug in the participant’s lifetime [25]. Besides, we also included specific types of drug use in the research analysis. It is worth mentioning that since drug use is an illegal activity, the number of people in the questionnaire who answered the item “number of days of drug use in the past 30 days” is limited, and we did not use this variable.

Sociodemographic characteristics included gender, age, race, marital status, income and education were assessed in the analysis. It is worth noting that the age and income variables were considered as skewed data, for which we transformed and performed as categorical variables. Gender was coded as either male or female. Age was divided into the 20–29 age group, 30–39 age group, 40–49 age group and 50–59 age group. The race was dichotomized into Hispanic, non-Hispanic White, African American and other race. Marital status classified as married, cohabiting couple, unmarried and the group consisting of widowed, divorced, separated. Income included lower-income (<= 4: $0–$1649), mediate-income (<= 8: $1650–$4599) and high-income (<=12: $4600 and over). Education was categorized as Less than high school (< high school), High school and some college (<= college) and college graduate or above (> college). Anxious days and sleep time were also assessed by questionnaire as additional potential covariables in predicting depression. Sleep time on the night was categorized into less than 6 h, 6–8 h and more than 8 h. Anxious days was assessed by the question: “during the past 30 days, for about how many days do you felt worried, tense, or anxious?” Using responses to this question, we classified respondents into the following groups: never, less than 1 week, one to 2 weeks, two to 3 weeks and over 3 weeks.

### Statistical analysis

Categorical data were shown as frequencies and proportions and compared by the Chi-square test. Multivariable logistic regression analysis was used to evaluate the independent risk factors for depression. We assessed associations between the predictors and the outcome of the resulting models using the odds ratios (OR). The nomogram is based on proportionally converting each regression coefficient in multivariable logistic regression to a 0 to a 100-point scale. The effect of the variable with the highest β coefficient (absolute value) is assigned 100 points. The points are added across independent variables to derive total points, which are converted to predicted probabilities [26]. Then, we developed the nomogram 1 according to the results of logistic regression. To further improve the predictive efficacy of the prediction model, we developed the nomogram 2 based on the nomogram 1. The model 1 (used for the nomogram 1) included gender, age, income, education, marital status, sleep time and illicit drug use, and the model 2 (used for the nomogram 2), furthermore, included anxious days. Calibration curves and a relatively corrected Harrell’s C-index was used to measure the prediction performance of nomograms. Given the possible impact of sampling weights of NHANES on the prediction model, 1000 cohorts with the same number of training cohort were generated by weighted random sampling and the area under the receiver operating characteristic curve (AUC) value of prediction model constructed by each cohort was calculated separately to evaluate the stability of the model. The net reclassification improvement (NRI) and integrated discrimination improvement (IDI) were calculated to estimate the discrimination or accuracy of the prediction models. We used the decision curve analysis (DCA) method to find a model to predict the maximum net benefit [27]. The statistical analyses were conducted with R software (Version 3.6.3, http://www.r-project.org/) and the R package glmnet (Version 4.1.3), riskRegression (Version 2021.10.10), pROC (Version 18.1.0), rms (Version 6.2.0), rmda (Version 1.6) and PredictABEL (Version 12.4), and *p* < 0.05 was considered significant.

## Results

The comparison of baseline characteristics of the depression and non-depression participants in the training cohort are listed in Table 1. The prevalence of depression was found in 841 (14.0%) and 373 (13.3%) in the training and validation cohort, respectively. The results showed that the prevalence of depression was significantly higher in female (57.1%) than in male (42.9%) in the training cohort (*P* < 0.001). And the participants in the 50–59 age group had a higher risk of depression (*P* < 0.001). We found that the risk of depression for participants with an education level below high school (29.8%) was higher than that for non-depression (15.2%), but participants with a college graduate or above (16.1%) had a significantly lower depression risk than non-depression (30.7%). Besides, participants with depression in the training cohort had a widowed, divorced or separated marriage (22.8%), earning less than $1649 a month (50.1%), sleep less than 6 h (25.7%), and worrying for more than 3 weeks (43.2%). The same demographic characteristics of depression are also shown in the validation cohort, as shown in eTable 1 (Data Supplement). The baseline characteristics of the training cohort and validation cohort participants are compared in eTable 2 (Data Supplement).

**Table 1:** Descriptive statistics of the study population in depression among the training cohort, NHANES, 2007–2010

Factors	Levels	Overall (*n* = 6015), %	Depression (*n* = 841), %	Non-depression (*n* = 5174), %	*P* value
Gender	Male	2991 (49.7)	344 (40.9)	2647 (51.2)	< 0.001
	Female	3024 (50.3)	497 (59.1)	2527 (48.8)	
Age	20–29	1464 (24.3)	169 (20.1)	1295 (25.0)	< 0.001
	30–39	1511 (25.1)	191 (22.7)	1320 (25.5)	
	40–49	1563 (26.0)	238 (28.3)	1325 (25.6)	
	50–59	1477 (24.6)	243 (28.9)	1234 (23.9)	
Race	Hispanic	1801 (29.9)	237 (28.2)	1564 (30.2)	0.033
	Non-Hispanic White	2800 (46.6)	374 (44.5)	2426 (46.9)	
	African American	1148 (19.1)	191 (22.7)	957 (18.5)	
	Other	266 (4.4)	39 (4.6)	227 (4.4)	
Marital status	Married	3027 (50.3)	319 (37.9)	2708 (52.3)	< 0.001
	Cohabiting couple	637 (10.6)	90 (10.7)	547 (10.6)	
	Unmarried	1360 (22.6)	216 (25.7)	1144 (22.1)	
	Wid/Div/Sep	991 (16.5)	216 (25.7)	775 (15.0)	
Education	< high school	1496 (24.9)	308 (36.6)	1188 (23.0)	< 0.001
	<= college	3246 (54.0)	449 (53.4)	2797 (54.1)	
	> college	1273 (21.2)	84 (10.0)	1189 (23.0)	
Income	<= 4	1881 (31.3)	399 (47.4)	1482 (28.6)	< 0.001
	<= 8	2349 (39.1)	322 (38.3)	2027 (39.2)	
	<= 12	1785 (29.7)	120 (14.3)	1665 (32.2)	
Sleep time	< 6 h	982 (16.3)	241 (28.7)	741 (14.3)	< 0.001
	<= 8 h	4673 (77.7)	527 (62.7)	4146 (80.1)	
	> 8 h	360 (6.0)	73 (8.7)	287 (5.5)	
Anxious day	Never	2269 (37.7)	72 (8.6)	2197 (42.5)	< 0.001
	<= 7 days	2117 (35.2)	177 (21.0)	1940 (37.5)	
	<= 14 days	470 (7.8)	76 (9.0)	394 (7.6)	
	<= 21 days	429 (7.1)	129 (15.3)	300 (5.8)	
	> 21 days	730 (12.1)	387 (46.0)	343 (6.6)	

Abbreviations: *NHANES* National Health and Nutrition Examination Survey, *Wid* Widowed, Div Divorced, *Sep* Separated; sleep time - How much sleep do you usually get at night on weekdays or workdays? Anxious day – during the past 30 days, for about how many days do you felt worried, tense, or anxious? Education was categorized as Less than high school (< high school), High school and some college (<= college) and college graduate or above (> college). Income included lower income (<= 4: $0–$1649), mediate income (<= 8: $1650–$4599) and high income (<=12: $4600 and over)

More than half (56.0%) of the sample reported using drugs at least once in their lifetime, especially marijuana (53.0%). About 20% of participants reported having used cocaine and illicit drug use in their lifetime. The proportion of heroin, methamphetamine and injecting drugs used in the sample population was 7.2, 3.2 and 2.8%, respectively. Statistical differences in methamphetamine use (29.33%), injecting drug use (27.86%), marijuana use (15.20%), drug use (15.56%), and illicit drug use (19.81%) between the depression and non-depression in the training cohort. However, no differences were found in heroin (*P* = 0.14) and cocaine (*P* = 0.79) use among the depression and non-depression (eTable 3, Data Supplement).

The results of multivariable logistic analysis with depression as the dependent variable are shown in Table 2. For the model 1, with results reported as odds ratio [95% CI], income (1.61[1.42–1.64]), female (1.66[1.42–1.94]), growing age (for 30–39 age group, 1.32[1.04–1.68]; for 40–49 age group, 1.53[1.2–1.95]; for 50–59 age group, 1.73[1.35–2.21]), lower educational level (for <= college, 0.65[0.55–0.78]; for > college, 0.39[0.29–0.51]), unmarried (1.48[1.19–1.84]),the group consisting of widowed, divorced and separated (1.38[1.12–1.7]), fewer sleep hours (0.86[0.82–0.9]) and illicit drug use (1.67[1.41–1.99]) were independently associated with depression. Furthermore, anxious days added to model 2 was found to be significantly associated with depression (for <= 7 days, 2.86[2.15–3.8]; for <= 14 days, 5.3[3.74–7.5]; for <= 21 days, 12.49[9.04–17.26]; for > 21 days, 30.97[23.28–41.21]).

**Table 2:** Potential associations between predictors in two models and depression

Predictors	Coding	Model 1 OR (95% CI)	Model 2 OR (95% CI)
Income	1–12 grade (ref = 12)	0.88(0.85–0.9)*	0.91(0.88–0.93)*
Gender	Female (ref = Male)	1.66(1.42–1.94)*	1..35(1.13–1.62)*
Age	30–39 (ref = 20–29)	1.32(1.04–1.68)*	1.16(0.89–1.51)
Age	40–49 (ref = 20–29)	1.53(1.2–1.95)*	1.32(1.01–1.72)*
Age	50–59 (ref = 20–29)	1.73(1.35–2.21)*	1.58(1.2–2.08)*
Education	<= college (ref = < high school)	0.65(0.55–0.78)*	0.65(0.53–0.79)*
Education	> college (ref = < high school)	0.39(0.29–0.51)*	0.36(0.27–0.48)*
Marital status	Cohabiting couple (ref = Married)	1.06(0.81–1.39)	1.02(0.75–1.38)
Marital status	Unmarried (ref = Married)	1.48(1.19–1.84)*	1.82(1.43–2.32)*
Marital status	Wid/Div/Sep (ref = Married)	1.38(1.12–1.7)*	1.39(1.09–1.76)*
Sleep time	1–12 h (ref = 12 h)	0.86(0.82–0.9)*	0.94(0.88–0.99)*
Illicit drug	Yes-No (ref = No)	1.67(1.41–1.99)*	1.38(1.13–1.67)*
Anxious day	<= 7 days (ref = Never)		2.86(2.15–3.8)*
Anxious day	<= 14 days (ref = Never)		5.3(3.74–7.5)*
Anxious day	<= 21 days (ref = Never)		12.49(9.04–17.26)*
Anxious day	> 21 days (ref = Never)		30.97(23.28–41.21)*

Note: *p<0.05

**Fig. 1:**
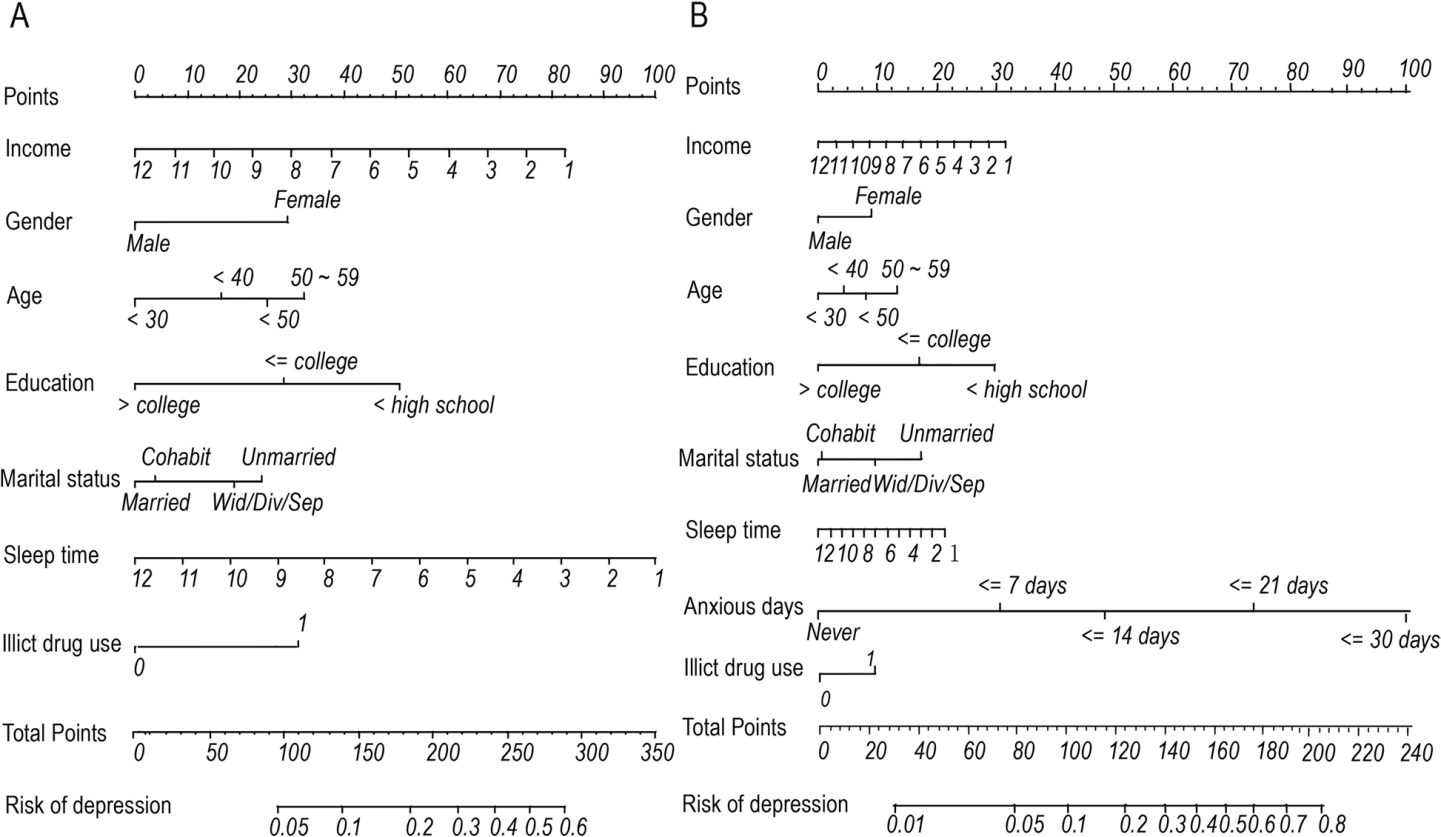
Nomogram to estimate the probability of depression risk. (**A**), The model 1 nomogram was developed in the training cohort, with income, gender, age, education, marital status, sleep time and illicit drug use. (**B**), The model 2 nomogram selected predictors were identical to the model 1, and the additional variables of anxious days indicators

**Fig. 2:**
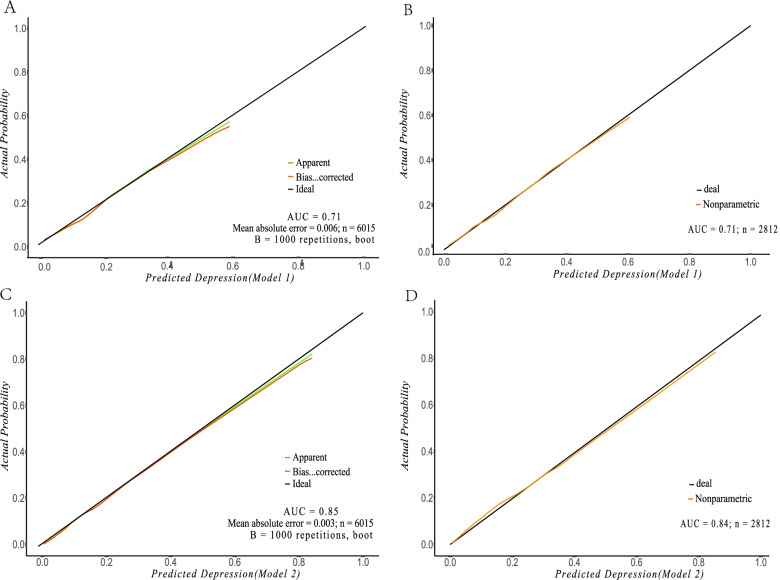
Calibration curves of the model 1 and 2 nomogram in the training cohort and validation cohort. (**A**), Calibration curve of the model 1 in the training cohort. (**B**), Calibration curve of the model 1 in the validation cohort. (**C**), Calibration curve of the model 2 in the training cohort. (**D**), Calibration curve of the model 2 in the validation cohort. The x-axis and y-axis represent the predicted risk of depression and the actual incidence of depression, respectively. The closer the black solid line and the black dotted line fit on the diagonal, the better the prediction effect

Based on the multivariable logistic regression results, 2 nomograms were developed and presented (shown in Fig. 1). Model 1 was effective in predicting depression at moderate-low risk populations, and model 2 was better in diagnosing high-risk depression populations. The validation results showed that there were four good calibration curves for risk estimation of depression (shown in Fig. 2). The prediction nomogram of model 1 yielded a bootstrap-corrected C index was 0.71 (95% CI, 0.69–0.73). And the nomogram of model 1 displayed a C-index of 0.71 (95% CI, 0.68–0.74) in the validation cohort. A bootstrap adjusted C-index for the prediction nomogram 2 was 0.85 (95% CI, 0.83–0.86). In the validation cohort, the nomogram 2 showed a C-index of 0.83 (95% CI, 0.81–0.86) for the estimation of depression risk. We calculate the AUC (the area under the receiver operating characteristic curve) mean and 95%CI of the prediction model under 1000 different weighted random sampling of model 1 (0.71[0.67–0.75])and model 2 (0.83[0.80–0.87]), respectively (eTable 4, Data Supplement). eTable 4 also showed that the AUC value (0.88–0.91) of the predictive model performed well under the depression scores of 10–17.

**Fig. 3:**
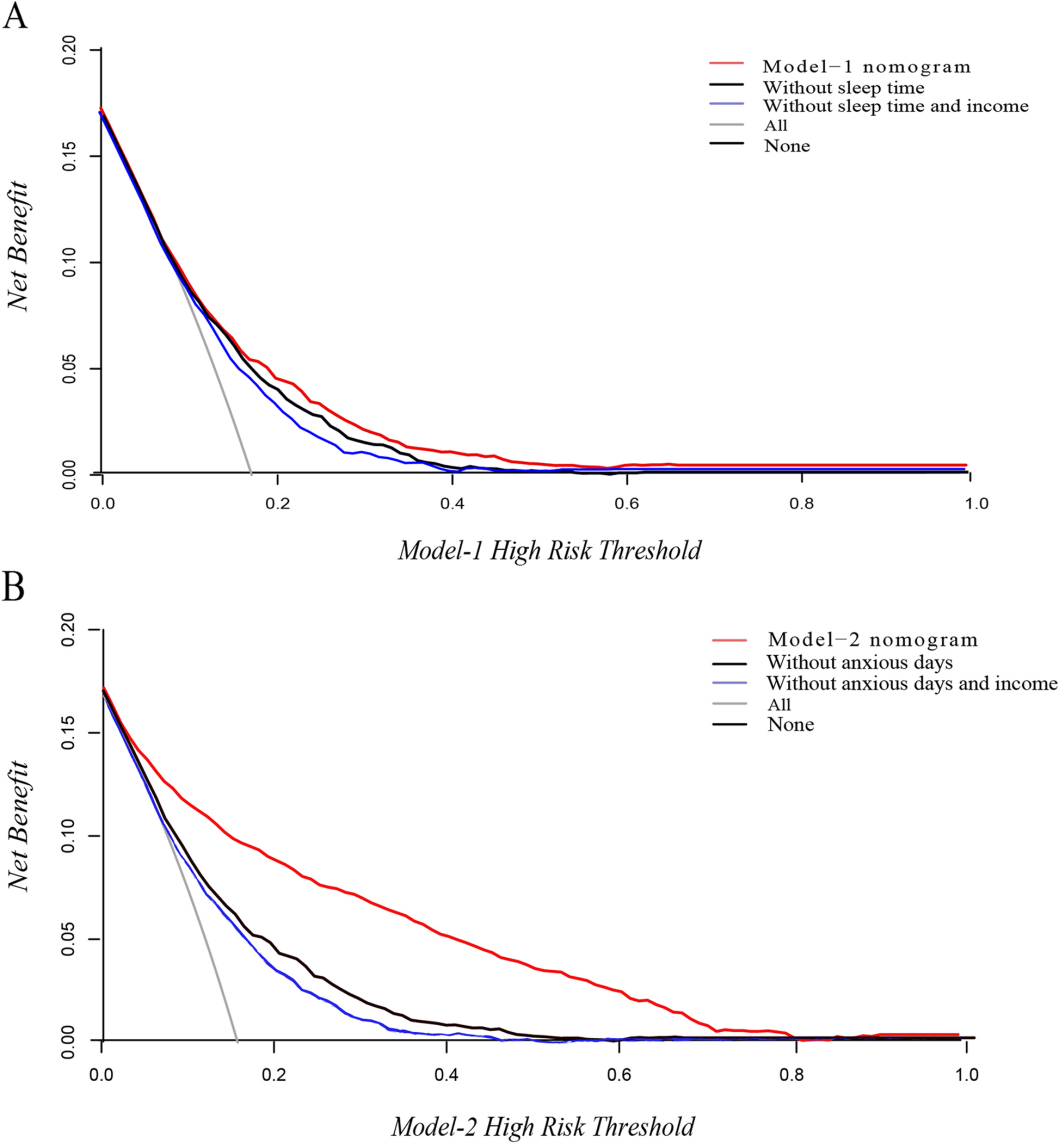
Decision curve analysis for the model 1 and model 2 nomogram. (**A**), The red line represents the model 1. The black line represents the assumption of model 1 without the variable sleep time. The blue line represents the assumption of model 1 that removes both sleep time and income variables. (**B**), The red line represents the model 2. The black line represents the assumption of model 2 without the variable number of anxious days. The blue line represents the assumption of model 2 that removes both anxious days and income variables. The x-axis represents the threshold probability of depression risk for participants, which we set at 16%. The y-axis measures the net benefit

In model 1, we found that income and sleep time improved the reclassification performance (NRI, 0.08[95%CI, 0.05–0.12]; *P* < 0.001); in model 2, the NRI with anxious days discriminated very well (NRI, 0.56[95%CI, 0.51–0.61]; *P* < 0.001). From the calculation results of IDI, the prediction probability of model 1 is 0.03 (95%, 0.02–0.03; *P* < 0.001) higher than that of the model 1 without income and sleep time. In model 2, we found that adding anxious days variables can develop the performance of the model 2 than the model 2 without anxious days variables (IDI, 0.19[95%CI, 0.18–0.20]; *P* < 0.001). In Fig. 3-A, the model 1 is superior to the other two models in the range of threshold probability of 0.2–0.4 and has a positive net benefit. Similarly, model 2 produces the maximum net benefit across almost threshold probability range, compared with the model without anxious days and income and the model without anxious days in Fig. 3-B. Especially at the 30% risk threshold, the difference in net benefit between model 2 and the other two models was 0.07 and 0.08, which is equivalent to detecting 7 and 8 more high-risk depression per 100 patients in the same number of depressions predicting samples. Furthermore, the web calculators can achieve the prediction results of depression risk in detail, as well as the dynamic process of prediction probability. (for model 1, https://hmuhan157-account.shinyapps.io/Depression-predicted-model1/; for model 2, https://hmuhan157-account.shinyapps.io/Depression-predicted-model2/).

## Discussion

In this study, approximately 20.0% of the drug users had a higher prevalence of depression than the general population (14.0%) and drug users had a significantly higher risk of depression than non-drug users (more than 1.5 times). Individuals at risk for depression were identified by adding demographic factors, sleep time, illicit drug use and anxious days. In particular, we found that anxious days have a wide range of values and have good applicability in predicting the high risk of depression.

The related risk factors into an easy-to-use nomogram facilitate the clinicians and patients facing the challenges of treating depression. Of course, with the rise of clinical prediction models, they have become more widely used in various fields, and their use in predicting depression has gradually increased. But we found that it was either limited by the small sample size, poor performance in the validation of depression models, or by the validation results, no external validation was found or the validation was only able to predict moderate-to-low-risk depression in multiple literature searches [28, 6, 29]. To our knowledge, few studies have placed illicit drug use in predictive models of depression risk.

Compared with other models, we identified demographics, sleep time and illicit drug use factors to determine depression risk models with good discriminative ability. In our model 1, demographic variables including female, age between 50 and 59 years, the educational level below high school, unmarried and lower-income were found to be associated with depression, which is in line with other studies [7, 5, 9]. Moreover, it is found that people with low education level and economic poverty more likely to use drugs [25, 30]. This explains to a certain extent that these demographic factors are more effective in the depression model. There is ample evidence of a link between sleep disorders and depression [31, 32]. The more important result for this study is the performance of internal and external validation of the model. We found that the ability of model 1 to distinguish between individuals with depression and those without depression was greater than 0.7, and the predicted probability of depression is aligned with the observed probability of depression along a 45-degree diagonal line. Moreover, the calculated resampling weight discrimination results show that the model is suitable for the U.S. civilian population and its representativeness and availability is one of the contributions of this study. Considering that sleep time and income had greater action on model 1, we examined the combined effect of these two factors on the model and found that the prediction accuracy and a prediction improvement and the positive net benefit were limited. It is regrettable that the model also can only predict the risk of moderate depression.

To better identify people at high-risk for predicting depression, we added the number of anxious days in the past 30 days to model 2. For model 2, the longer the anxious days lasted, the higher the risk of depression. Although depression and anxiety often appear together, the hallmark symptoms of depression are emotional (hopelessness and sadness), the highest scores found were physical: appetite, fatigue, and poor sleep, whereas anxiety tends to be overly fearful, referring to perceived anticipated threat [33, 34]. Given the correlation with outcomes, we used only anxious days, a relatively objective indicator, to predict depression risk. The discrimination of model 2 was greater than 0.8 and the calibration curve performed well. The reclassification improvement of model 2 by anxious days was improved by 0.56, the prediction accuracy of model 2 was improved by 0.19, and the predicted net benefit obtained by model 2 was improved by 0.07. Model 2 not only has an advantage in predicting high-risk groups but also can be reliably applied to the general population. Therefore, we conclude that measures of anxious days had a higher and more specific predictive power for depression at high-risk, suggesting that we should focus on the anxious days in patients, which has an important value in improving the depression.

Although PHQ-9 is an effective tool for depression screening, the criteria determined by the PHQ-9 scale may be biased in comparison with clinical diagnosis [35]. We reconstructed the models based on model 2 by extending the range of the depression threshold and calculated the discrimination of these models. Encouragingly, as depression scores increased, these models perform better in identifying people at high risk for depression. Besides, network calculators provide a convenient tool for quickly and visually assessing the performance of prediction models.

The current study is subject to several limitations. Firstly, it still needs to be verified in a large sample of different countries to determine the performance of the prediction model in the cross-cultural context. Secondly, this study was a cross-sectional design, so further prospective study design needs to trace the causal relationship. Thirdly, limited by the lack of variables in the database, in the future we should include predictor variables such as somatic illnesses. Fourthly, although the prediction model performs very well in discrimination and calibration but remains false-positive and false-negative rates in models. Finally, given the correlation between anxious days and depression outcomes, additional, objective indicators such as biomarker markers need to be considered to identify the high-risk population.

## Conclusions

In conclusion, we construct two models for depression by combining the predictive factors of gender, age, income, education, marital status, sleep time, illicit drug use and anxious days. In particular, the identified anxious days in predicting high-risk depression had good discriminative ability compared with model 1. The study provides an optimal estimation of the predicted probability of depression risk.

## Supplementary Information

**Additional file 1: eTable 1.** Descriptive statistics of the study population in depression among the validation cohort, NHANES, 2011–2012. eTable 2. Descriptive statistics of the study population among the training cohort and validation cohort, NHANES, 2007–2012. eTable 3. Drug use and depression among the training cohort, NHANES, 2007–2010, (*n* = 7076). eTable 4. Evaluation of the differential diagnosis ability of predictive models under different weighted random sampling and different diagnostic criteria for depression in the validation cohort. eFig. 1. Flow diagram of the study participants.

## Abbreviations

NHANES: National Health and Nutrition Examination Survey
PHQ-9: Patient Health Questionnaire-9
DUQ: Drug use questionnaire
AUC: The area under the receiver operating characteristic curve
NRI: Net reclassification improvement
IDI: Integrated discrimination improvement

## References

[1] Richards DA, Ekers D, McMillan D, Taylor RS, Byford S, Warren FC (2016). Cost and Outcome of Behavioural Activation versus Cognitive Behavioural Therapy for Depression (COBRA): a randomised, controlled, non-inferiority trial. Lancet..

[2] American Psychiatric Association (2013). Diagnostic and Statistical Manual of Mental Disorders.

[3] James SL, Abate D, Abate KH, Abay SM, Abbafati C, Abbasi N (2018). Global, regional, and national incidence, prevalence, and years lived with disability for 354 diseases and injuries for 195 countries and territories, 1990–2017: a systematic analysis for the Global Burden of Disease Study 2017. Lancet..

[4] Malhi GS, Mann JJ (2018). Depression. Lancet..

[5] Argent V, Smyth RS (2019). Editorial: is cirrhosis depressing?. Aliment Pharmacol Ther..

[6] Jia Y, Zhang W, You S, Li M, Lei L, Chen L (2019). A nomogram for predicting depression in patients with hepatocellular carcinoma: an observational cross-sectional study. Int J Clin Pract..

[7] Salk RH, Hyde JS, Abramson LY (2017). Gender differences in depression in representative national samples: Meta-analyses of diagnoses and symptoms. Psychol Bull..

[8] Williams DR, González HM, Neighbors H, Nesse R, Abelson JM, Sweetman J (2007). Prevalence and distribution of major depressive disorder in African Americans, Caribbean blacks, and non-Hispanic whites: results from the National Survey of American Life. Arch Gen Psychiatry..

[9] Hakulinen C, Musliner KL, Agerbo E (2019). Bipolar disorder and depression in early adulthood and long-term employment, income, and educational attainment: A nationwide cohort study of 2,390,127 individuals. Depress Anxiety..

[10] Erickson J, El-Gabalawy R, Palitsky D, Patten S, Mackenzie CS, Stein MB (2016). Educational attainment as a protective factor for psychiatric disorders: findings from a nationally representative longitudinal study. Depress Anxiety..

[11] Auerbach RP, Mortier P, Bruffaerts R, Alonso J, Benjet C, Cuijpers P (2018). WHO World Mental Health Surveys International College Student Project: Prevalence and distribution of mental disorders. J Abnorm Psychol..

[12] Christie-Mizell CA, Talbert RD, Hope AR, Frazier CG, Hearne BN (2019). Depression and African Americans in the First Decade of Midlife: The Consequences of Social Roles and Gender. J Natl Med Assoc..

[13] Strohschein L, McDonough P, Monette G, Shao Q (2005). Marital transitions and mental health: are there gender differences in the short-term effects of marital status change?. Soc Sci Med..

[14] Chellappa SL, Schröder C, Cajochen C (2009). Chronobiology, excessive daytime sleepiness and depression: Is there a link?. Sleep Med..

[15] Fava M (2004). Daytime sleepiness and insomnia as correlates of depression. J Clin Psychiatry..

[16] Difrancesco S, Lamers F, Riese H, Merikangas KR, Beekman ATF, van Hemert AM (2019). Sleep, circadian rhythm, and physical activity patterns in depressive and anxiety disorders: A 2-week ambulatory assessment study. Depress Anxiety..

[17] Beard C, Millner AJ, Forgeard MJ, Fried EI, Hsu KJ, Treadway MT (2016). Network analysis of depression and anxiety symptom relationships in a psychiatric sample. Psychol Med..

[18] Gobbi G, Atkin T, Zytynski T, Wang S, Askari S, Boruff J (2019). Association of Cannabis Use in Adolescence and Risk of Depression, Anxiety, and Suicidality in Young Adulthood: A Systematic Review and Meta-analysis. JAMA Psychiatry..

[19] Preece RL, Han SYS, Bahn S (2018). Proteomic approaches to identify blood-based biomarkers for depression and bipolar disorders. Expert Rev Proteomics..

[20] Liu B, Sun Y, Xu G, Du Y, Ajjarapu AS, Snetselaar LG (2019). Association between plasma concentrations of elaidic acid, a major trans fatty acid, and depression in a nationally representative sample of U.S. adults. J Affect Disord..

[21] Manea L, Gilbody S, McMillan D (2015). A diagnostic meta-analysis of the Patient Health Questionnaire-9 (PHQ-9) algorithm scoring method as a screen for depression. Gen Hosp Psychiatry..

[22] Manea L, Gilbody S, McMillan D (2012). Optimal cut-off score for diagnosing depression with the Patient Health Questionnaire (PHQ-9): a meta-analysis. Can Med Assoc J..

[23] Kroenke K, Spitzer RL, Williams JB, Löwe B (2010). The Patient Health Questionnaire Somatic, Anxiety, and Depressive Symptom Scales: a systematic review. Gen Hosp Psychiatry..

[24] Patel JS, Oh Y, Rand KL, Wu W, Cyders MA, Kroenke K (2019). Measurement invariance of the patient health questionnaire-9 (PHQ-9) depression screener in U.S. adults across sex, race/ethnicity, and education level: NHANES 2005–2016. Depress Anxiety..

[25] Vidot DC, Arheart KL, Prado G, Bandstra ES, Messiah SE (2013). Illicit drug use and cardiometabolic disease risk: an analysis of 2005–2008 National Health and Nutrition Examination Survey data. Int J Clin Pract..

[26] Lei Z, Li J, Wu D, Xia Y, Wang Q, Si A (2016). Nomogram for Preoperative Estimation of Microvascular Invasion Risk in Hepatitis B Virus-Related Hepatocellular Carcinoma Within the Milan Criteria. JAMA Surg..

[27] Van Calster B, Wynants L, Verbeek JFM, Verbakel JY, Christodoulou E, Vickers AJ (2018). Reporting and Interpreting Decision Curve Analysis: A Guide for Investigators. Eur Urol..

[28] Buganza-Torio E, Mitchell N, Abraldes JG, Thomas L, Ma M, Bailey RJ (2019). Depression in cirrhosis - a prospective evaluation of the prevalence, predictors and development of a screening nomogram. Aliment Pharmacol Ther..

[29] Li G, Mei J, You J, Miao J, Song X, Sun W (2019). Sociodemographic characteristics associated with adolescent depression in urban and rural areas of Hubei province: a cross-sectional analysis. BMC Psychol..

[30] Kurani S, McCoy RG, Inselman J, Jeffery MM, Chawla S, Finney Rutten LJ (2020). Place, poverty and prescriptions: a cross-sectional study using Area Deprivation Index to assess opioid use and drug-poisoning mortality in the USA from 2012 to 2017. BMJ Open..

[31] Dauvilliers Y, Lopez R, Ohayon M, Bayard S (2013). Hypersomnia and depressive symptoms: methodological and clinical aspects. BMC Med..

[32] Lopez R, Barateau L, Evangelista E, Dauvilliers Y (2017). Depression and Hypersomnia: A Complex Association. J Clin Sleep Med..

[33] Peres MFP, Mercante JPP, Tobo PR, Kamei H, Bigal ME (2017). Anxiety and depression symptoms and migraine: a symptom-based approach research. J Headache Pain..

[34] World Health Organization (2018). International Classification of Diseases Web site.

[35] Hartung TJ, Friedrich M, Johansen C, Wittchen HU, Faller H, Koch U (2017). The Hospital Anxiety and Depression Scale (HADS) and the 9-item Patient Health Questionnaire (PHQ-9) as screening instruments for depression in patients with cancer. Cancer..

