# Supplementary material for: Assessing the depression risk in the U.S. adults using nomogram
Source: BMC Public Health. 2022 Mar 2;22:416. doi: 10.1186/s12889-022-12798-6 (PMC8889727; doi:10.1186/s12889-022-12798-6)
Supplement: Supplementary file 1 — Additional file 1: eTable 1. Descriptive statistics of the study population in depression among the validation cohort, NHANES, 2011–2012. eTable 2. Descriptive statistics of the study population among the training cohort and validation cohort, NHANES, 2007–2012. eTable 3. Drug use and depression among the training cohort, NHANES, 2007–2010, (n = 7076). eTable 4. Evaluation of the differential diagnosis ability of predictive models under different weighted random sampling and different diagnostic criteria for depression in the validation cohort. eFig. 1. Flow diagram of the study participants. [file 12889_2022_12798_MOESM1_ESM.docx]

eTable 1 Descriptive statistics of the study population in depression among the validation cohort, NHANES, 2011–2012.

Abbreviations: NHANES - National Health and Nutrition Examination Survey; Wid – Widowed; Div – Divorced; Sep – Separated; sleep time - How much sleep do you usually get at night on weekdays or workdays? Anxious day – during the past 30 days, for about how many days do you felt worried, tense, or anxious?

Education was categorized as Less than high school (< high school), High school and some college (<= college) and college graduate or above (> college). Income included lower income (<= 4: $0-$1649), mediate income (<= 8: $1650-$4599) and high income (<=12: $4600 and over).

| Factors | Levels | Overall  (n = 2812), % | Depression  (n = 373), % | Non-depression  (n = 2439), % | P value |
| --- | --- | --- | --- | --- | --- |
| Gender | Male | 1450 (51.6) | 160 (42.9) | 1290 (52.9) | <0.001 |
|  | Female | 1362 (48.4) | 213 (57.1) | 1149 (47.1) |  |
| Age | 20 ~ 29 | 749 (26.6) | 76 (20.4) | 673 (27.6) | <0.001 |
|  | 30 ~ 39 | 687 (24.4) | 77 (20.6) | 610 (25.0) |  |
|  | 40 ~ 49 | 662 (23.5) | 96 (25.7) | 566 (23.2) |  |
|  | 50 ~ 59 | 714 (25.4) | 124 (33.2) | 590 (24.2) |  |
| Race | Hispanic | 547 (19.5) | 68 (18.2) | 479 (19.6) | 0.027 |
|  | Non-Hispanic White | 1072 (38.1) | 146 (39.1) | 926 (38.0) |  |
|  | African American | 729 (25.9) | 114 (30.6) | 615 (25.2) |  |
|  | Other | 464 (16.5) | 45 (12.1) | 419 (17.2) |  |
| Education | < high school | 482 (17.1) | 111 (29.8) | 371 (15.2) | <0.001 |
|  | <= college | 1521 (54.1) | 202 (54.2) | 1319 (54.1) |  |
|  | > college | 809 (28.8) | 60 (16.1) | 749 (30.7) |  |
| Marital status | Married | 1288 (45.8) | 114 (30.6) | 1174 (48.1) | <0.001 |
|  | Cohabiting couple | 293 (10.4) | 49 (13.1) | 244 (10.0) |  |
|  | Unmarried | 809 (28.8) | 125 (33.5) | 684 (28.0) |  |
|  | Wid/Div/Sep | 422 (15.0) | 85 (22.8) | 337 (13.8) |  |
| Income | <= 4 | 893 (31.8) | 187 (50.1) | 706 (28.9) | <0.001 |
|  | <= 8 | 1061 (37.7) | 139 (37.3) | 922 (37.8) |  |
|  | <= 12 | 858 (30.5) | 47 (12.6) | 811 (33.3) |  |
| Sleep time | < 6 hours | 471 (16.7) | 96 (25.7) | 375 (15.4) | <0.001 |
|  | <= 8 hours | 2162 (76.9) | 246 (66.0) | 1916 (78.6) |  |
|  | > 8 hours | 179 (6.4) | 31 (8.3) | 148 (6.1) |  |
| Anxious day | Never | 1219 (43.3) | 47 (12.6) | 1172 (48.1) | <0.001 |
|  | <= 7 days | 918 (32.6) | 75 (20.1) | 843 (34.6) |  |
|  | <= 14 days | 187 (6.7) | 37 (9.9) | 150 (6.2) |  |
|  | <= 21 days | 182 (6.5) | 53 (14.2) | 129 (5.3) |  |
|  | > 21 days | 306 (10.9) | 161 (43.2) | 145 (5.9) |  |

eTable 2 Descriptive statistics of the study population among the training cohort and validation cohort, NHANES, 2007–2012.

Abbreviations: NHANES - National Health and Nutrition Examination Survey; Wid – Widowed; Div – Divorced; Sep – separated; sleep time - How much sleep do you usually get at night on weekdays or workdays? Anxious day – during the past 30 days, for about how many days do you felt worried, tense, or anxious?

Education was categorized as Less than high school (< high school), High school and some college (<= college) and college graduate or above (> college). Income included lower income (<= 4: $0-$1649), mediate income (<= 8: $1650-$4599) and high income (<=12: $4600 and over).

| Factors | Levels | Overall  (n = 8827), % | Training  (n = 6015), % | Validation (n = 2812), % | P value |
| --- | --- | --- | --- | --- | --- |
| Gender | Male | 4441 (50.3) | 2991 (49.7) | 1450 (51.6) | 0.112 |
|  | Female | 4386 (49.7) | 3024 (50.3) | 1362 (48.4) |  |
| Age | 20 ~ 29 | 2213 (25.1) | 1464 (24.3) | 749 (26.6) | 0.024 |
|  | 30 ~ 39 | 2198 (24.9) | 1511 (25.1) | 687 (24.4) |  |
|  | 40 ~ 49 | 2225 (25.2) | 1563 (26.0) | 662 (23.5) |  |
|  | 50 ~ 59 | 2191 (24.8) | 1477 (24.6) | 714 (25.4) |  |
| Race | Hispanic | 2348 (26.6) | 1801 (29.9) | 547 (19.5) | <0.001 |
|  | Non-Hispanic White | 3872 (43.9) | 2800 (46.6) | 1072 (38.1) |  |
|  | African American | 1877 (21.3) | 1148 (19.1) | 729 (25.9) |  |
|  | Other | 730 (8.3) | 266 (4.4) | 464 (16.5) |  |
| Education | < high school | 1978 (22.4) | 1496 (24.9) | 482 (17.1) | <0.001 |
|  | <= college | 4767 (54.0) | 3246 (54.0) | 1521 (54.1) |  |
|  | > college | 2082 (23.6) | 1273 (21.2) | 809 (28.8) |  |
| Marital status | Married | 4315 (48.9) | 3027 (50.3) | 1288 (45.8) | <0.001 |
|  | Cohabiting couple | 930 (10.5) | 637 (10.6) | 293 (10.4) |  |
|  | Unmarried | 2169 (24.6) | 1360 (22.6) | 809 (28.8) |  |
|  | Wid/Div/Sep | 1413 (16.0) | 991 (16.5) | 422 (15.0) |  |
| Income | <= 4 | 2774 (31.4) | 1881 (31.3) | 893 (31.8) | 0.483 |
|  | <= 8 | 3410 (38.6) | 2349 (39.1) | 1061 (37.7) |  |
|  | <= 12 | 2643 (29.9) | 1785 (29.7) | 858 (30.5) |  |
| Sleep time | < 6 hours | 1453 (16.5) | 982 (16.3) | 471 (16.7) | 0.662 |
|  | <= 8 hours | 6835 (77.4) | 4673 (77.7) | 2162 (76.9) |  |
|  | > 8 hours | 539 (6.1) | 360 (6.0) | 179 (6.4) |  |
| Anxious day | Never | 3488 (39.5) | 2269 (37.7) | 1219 (43.3) | <0.001 |
|  | <= 7 days | 3035 (34.4) | 2117 (35.2) | 918 (32.6) |  |
|  | <= 14 days | 657 (7.4) | 470 (7.8) | 187 (6.7) |  |
|  | <= 21 days | 611 (6.9) | 429 (7.1) | 182 (6.5) |  |
|  | > 21 days | 1036 (11.7) | 730 (12.1) | 306 (10.9) |  |

eTable 3 Drug use and depression among the training cohort, NHANES, 2007–2010, (n = 7076).

| Factors | Overall  N (%) | Non-depression  N (%) | Depression  N (%) | P value |
| --- | --- | --- | --- | --- |
| Drug use | 3965(56.03) | 3348(84.44) | 617(15.56) | <0.01 |
| Illicit drug use | 1484(20.97) | 1190(80.19) | 294(19.81) | <0.01 |
| Ever used marijuana | 3751(53.01) | 3181(84.80) | 570(15.20) | <0.01 |
| Ever used cocaine | 1400(19.79) | 1129(80.64) | 271(19.56) | 0.14 |
| Ever used heroin | 508(7.18) | 405(79.72) | 103(20.28) | 0.73 |
| Ever used methamphetamine | 225(3.18) | 159(70.67) | 66(29.33) | <0.01 |
| Ever used inject drug | 201(2.84) | 145(72.14) | 56(27.86) | <0.01 |

eTable 4 Evaluation of the differential diagnosis ability of predictive models under different weighted random sampling and different diagnostic criteria for depression in the validation cohort.

| Factors | AUC value (95% CI) |
| --- | --- |
| The AUC mean value under 1000 weighted random sampling in Model 1 | 0.71(0.67~0.75) |
| The AUC mean value under 1000 weighted random sampling in Model 2 | 0.83(0.80~0.87) |
| The AUC value of the prediction model with a cut-off score of 10 in PHQ-9 | 0.88(0.85~0.90) |
| The AUC value of the prediction model 2 with a cut-off score of 11 in PHQ-9 | 0.88(0.86~0.91) |
| The AUC value of the prediction model 2 with a cut-off score of 12 in PHQ-9 | 0.89(0.87~0.92) |
| The AUC value of the prediction model 2 with a cut-off score of 13 in PHQ-9 | 0.89(0.86~0.92) |
| The AUC value of the prediction model 2 with a cut-off score of 14 in PHQ-9 | 0.88 (0.85~0.91) |
| The AUC value of the prediction model 2 with a cut-off score of 15 in PHQ-9 | 0.88(0.85~0.92) |
| The AUC value of the prediction model 2 with a cut-off score of 16 in PHQ-9 | 0.89(0.85~0.93) |
| The AUC value of the prediction model 2 with a cut-off score of 17 in PHQ-9 | 0.91(0.88~0.95) |

Abbreviations: AUC- the area under the receiver operating characteristic curve; PHQ-9 - the Patient

Health Questionnaire.


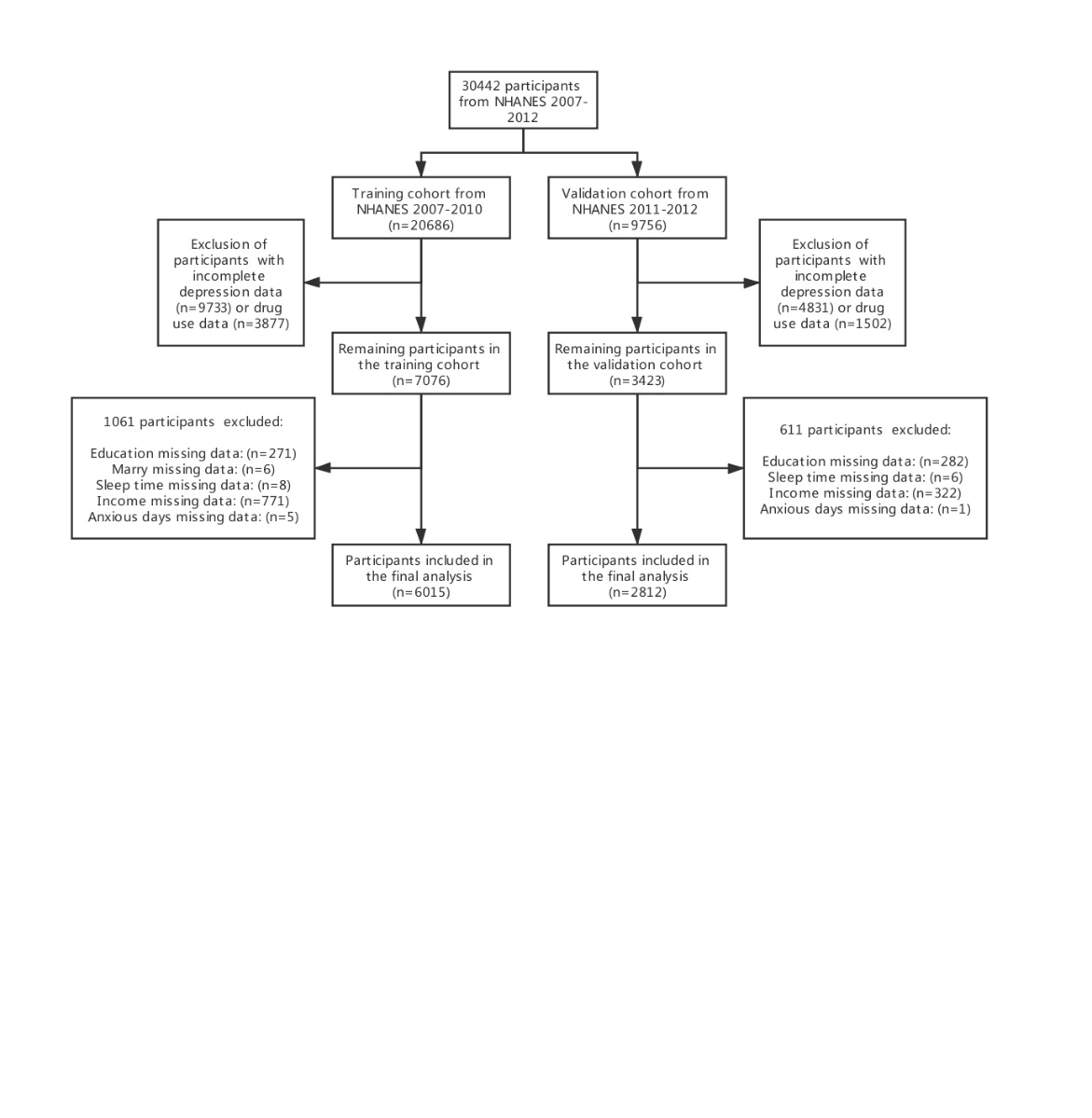


**eFig. 1. Flow diagram of the study participants.**
